# Women’s Awareness and Knowledge of Abortion Laws: A Systematic Review

**DOI:** 10.1371/journal.pone.0152224

**Published:** 2016-03-24

**Authors:** Anisa R. Assifi, Blair Berger, Özge Tunçalp, Rajat Khosla, Bela Ganatra

**Affiliations:** 1 WHO Department of Reproductive Health and Research, including UNDP/UNFPA/UNICEF/WHO/World Bank Special Programme of Research, Development and Research Training in Human Reproduction, World Health Organization, Geneva, Switzerland; 2 Department of Population, Family and Reproductive Health, Johns Hopkins University Bloomberg School of Public Health, Baltimore, Maryland, United States of America; University of Louisville School of Medicine, UNITED STATES

## Abstract

**Background:**

Incorrect knowledge of laws may affect how women enter the health system or seek services, and it likely contributes to the disconnect between official laws and practical applications of the laws that influence women’s access to safe, legal abortion services.

**Objective:**

To provide a synthesis of evidence of women’s awareness and knowledge of the legal status of abortion in their country, and the accuracy of women’s knowledge on specific legal grounds and restrictions outlined in a country’s abortion law.

**Methods:**

A systematic search was carried for articles published between 1980–2015. Quantitative, mixed-method data collection, and objectives related to women’s awareness or knowledge of the abortion law was included. Full texts were assessed, and data extraction done by a single reviewer. Final inclusion for analysis was assessed by two reviewers. The results were synthesised into tables, using narrative synthesis.

**Results:**

Of the original 3,126 articles, and 16 hand searched citations, 24 studies were included for analysis. Women’s correct general awareness and knowledge of the legal status was less than 50% in nine studies. In six studies, knowledge of legalization/liberalisation ranged between 32.3% - 68.2%. Correct knowledge of abortion on the grounds of rape ranged from 12.8% – 98%, while in the case of incest, ranged from 9.8% - 64.5%. Abortion on the grounds of fetal impairment and gestational limits, varied widely from 7% - 94% and 0% - 89.5% respectively.

**Conclusion:**

This systematic review synthesizes literature on women’s awareness and knowledge of the abortion law in their own context. The findings show that correct general awareness and knowledge of the abortion law and legal grounds and restrictions amongst women was limited, even in countries where the laws were liberal. Thus, interventions to disseminate accurate information on the legal context are necessary.

## Introduction

The World Health Organization (WHO) estimates that an annual 22 million unsafe abortions occur globally, almost all of which take place in developing countries [1]. Complications from unsafe abortion result in maternal deaths and abortion-related morbidities worldwide, placing high strain on limited health system resources and leading to severe physical, psychological, and financial consequences for women [2].

Barriers to accessing safe abortion exist at multiple levels. These include the legal and policy context of abortion, uneven access/distribution of safe and affordable abortion services, and insufficient capacity of provider cadres to provide high-quality services. Additionally, socioeconomic conditions, and stigma act as additional barriers to access [2]. Women’s own awareness of perception of legal status can influence their care seeking behaviour and ability to access safe and legal providers both in contexts where abortion is restrictive and in contexts where it is not. Even in countries with liberal abortion laws, women’s lack of awareness about the legal grounds on which abortion is permissible can prevent them from accessing services that may otherwise be available. Incorrect knowledge of laws may impact how women enter the health system or seek services, and it likely contributes to the disconnect between official laws and practical applications of the laws that influence women’s access to safe, legal abortion services [1, 2].

The proportion of women and health care providers with correct knowledge of legal status of abortion are both indicators for measuring access to information about safe abortion as outlined in the WHO guideline on safe abortion [1]. While there are systematic reviews focusing on healthcare providers’ attitudes and knowledge of abortion law in a specific country [3] or on their perceptions and attitudes towards providing abortions [4], there seems to be a gap in evidence synthesis for women’s knowledge of abortion laws. Therefore, our review aims to contribute to filling this critical gap and focuses on women’s awareness and knowledge of abortion legislation.

The specific objective of this systematic review is to provide a synthesis of evidence and summary of women’s knowledge in two main domains: 1) women’s awareness and knowledge of the legal status of abortion in their country, and 2) the accuracy of women’s knowledge on the specific legal grounds and restrictions outlined in a country’s abortion law.

## Methods

A protocol was developed in the process of this systematic review and is available on request.

### Search Strategy

A comprehensive systematic search of seven databases (PubMed, Popline, Medline, IBECS, MedCarib, BDENF-Nursing, and LILACS) was carried out in April 2014 and updated in March 2015. The three areas of focus of the search were: (1) abortion, (2) law, and (3) knowledge. A search strategy was developed for PubMed (S1 Table), using relevant medical subject headings (MeSH) and free-text words for each of the study components and was applied to the other databases accordingly. Search terms included a combination of abortion, legal, legislation, jurisprudence, awareness and knowledge. Nine test articles were used to determine the most comprehensive and efficient variation of search strategy. There were no language restrictions on the search.

The searches were restricted to identify articles published from January 1980 to the end of March 2015. The reference lists of all studies were also reviewed for additional relevant articles, including both unpublished and peer-reviewed papers. Though formal searches were not specifically conducted for grey literature or conference reports, if relevant articles and reports were found they were included.

### Study Selection

Articles were initially screened by one author (AA) based on title and abstract to determine if they met the inclusion criteria for full-text review. Criteria for inclusion were the following: (a) quantitative or mixed-method data collection, (b) study objectives related to awareness and/or knowledge of abortion law, and (c) study sample included women. For the purposes of this review, *awareness* was conceptualised as an individual being aware of a law i.e., knowing that an abortion law exists in a particular country. In contrast, *knowledge* was defined as an individual knowing the legal context and is able to identify the conditions under which abortion is legal. Studies were excluded if they only examined women’s attitudes toward abortion or opinions on abortion laws, or if the methodology or results were insufficiently reported to calculate proportions of correct awareness of knowledge. The review team were able to assess articles in English, French, and Turkish and a translation program was used for the articles in other languages and crosschecked if possible. If translation of an article written in a language other than English was not possible, the article was excluded.

### Data Extraction

A standardized form was developed to extract data in the following areas: 1) study setting and study population, 2) study methodology, 3) characteristics of the study population (e.g., age, marital status, urban/rural setting), 4) country’s abortion law at time of data collection, 5) results on proportions of correct awareness and/or knowledge, and 6) further notes/remarks. All articles were initially reviewed, and data extraction done by AA. Article review was not blinded to authorship of the studies. Final inclusion for analysis was assessed by two reviewers (AA and BB).

### Data Synthesis

Information from the included articles was compiled in a spreadsheet. The results were synthesised into tables based on the two objectives of the review. As the methods used to assess and determine level of knowledge, the study sampling frames and the populations studied varied across the included studies, the analysis was conducted using narrative synthesis.

As the description of the legal context extracted from each study varied, we used the United Nations Population Division (UNPD) description to categorise countries into three levels on the basis of their legal grounds for abortion at the time of the study [5, 6]. Countries were considered to have a *restrictive* abortion policy context if abortion was not allowed at all, to only preserve the life of the woman, and/or to preserve physical health. *Less restrictive* contexts were countries where abortion was allowed to preserve mental health and/or on socio-economic grounds. Finally, *least restrictive* countries were those where abortion was allowed on request [6].

## Results

**Fig 1:**
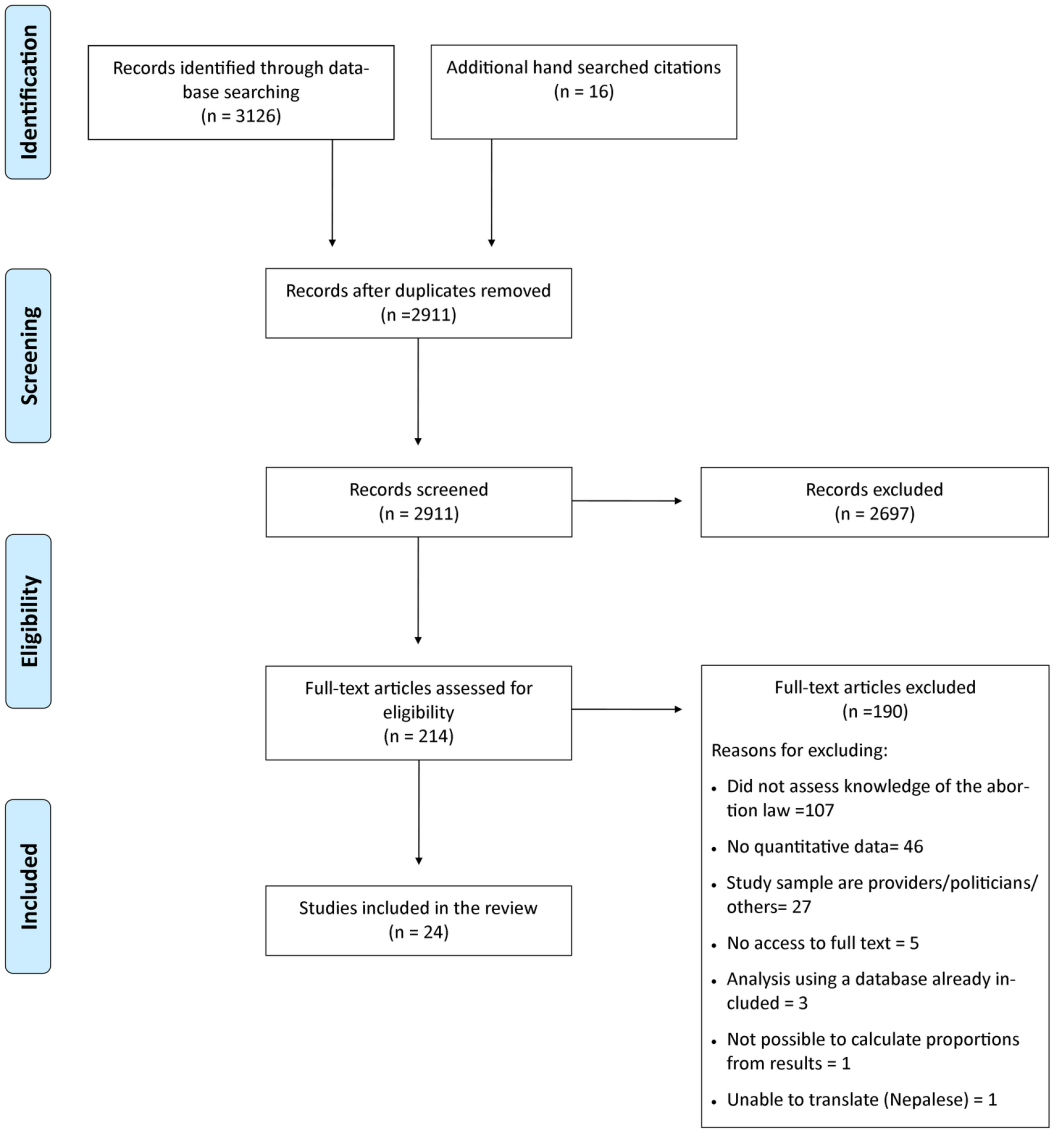
PRISMA Flow diagram of search and inclusion process.

The original search yielded 3,126 articles, and an additional 16 papers were found in a review of included articles’ reference lists. After excluding the duplicates, a total of 2,895 studies were screened for inclusion based on title and abstract, of which 213 underwent full-text review, and 24 studies were included for analysis (Fig 1).

**Fig 2:**
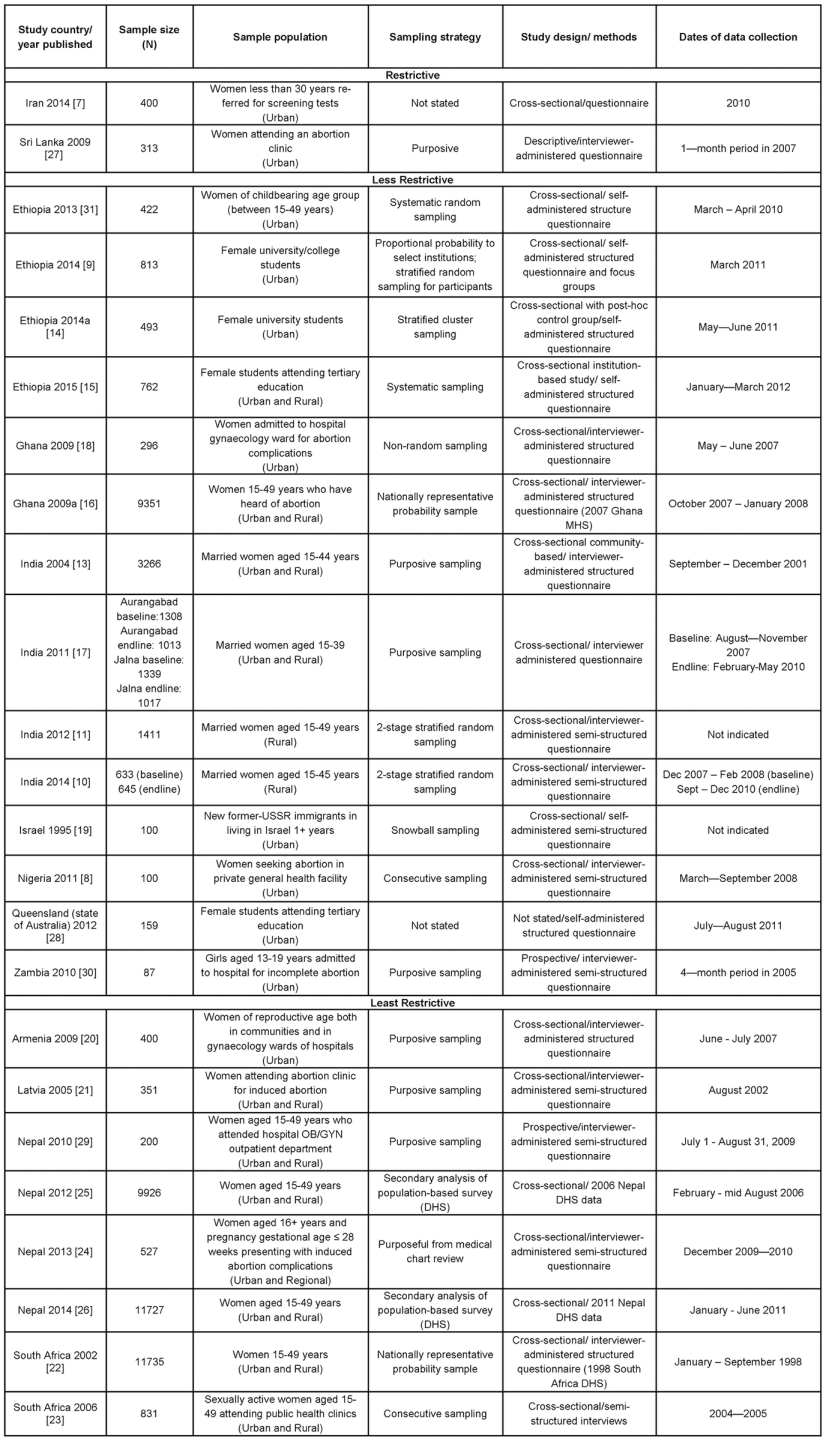
Characteristics of studies included (1995–2015) by restrictiveness of legal status—Women's knowledge (N = 24).

Fig 2 outlines the characteristics of the 24 studies included in this review. The majority were cross-sectional studies (22) [7–28], and two were prospective studies [29, 30]. The manuscripts were published between 1995 to 2015, and reported on data from 13 countries (majority low- and middle-income countries). Thirteen studies (54%) were published in 2011 to 2015 [7–11, 14, 15, 17, 24–26, 28], followed by 10 studies published between 2000 and 2010 [13, 16, 18, 20–23, 27, 29, 30]. Only one of the included studies was published before 2000 [19]. The sample sizes of the studies included ranged from 87 [30] to 11,735 women [22].

Based on the categorisation described above, only two studies (8%) were conducted in restrictive contexts (Sri Lanka and Iran) [7, 27], 14 (58%) were done in less restrictive contexts [8–19, 28, 30], and 8 (33%) were in least restrictive settings [20–26, 29]. None of the included studies were from the Latin American region. It should be noted that for the study conducted in Australia [28], because the study focused on the state of Queensland, which has a more restrictive policy context than the rest of Australia (not allowed on request), its policy context was categorized based on the state law.

Sampling strategies and questions posed to participants to determine awareness/knowledge showed considerable variation across studies. Four of the studies are nationally based samples [16, 22, 25, 26], and five are sub-national population samples of women [10, 11, 13, 17, 31]. For 10 of the studies, the study sample was taken from a health facility setting (hospital or clinic) [7, 8, 18, 20, 21, 23, 24, 27, 29, 30]. Three of the studies sampled women who had presented for incomplete abortions [18, 24, 30], 3 other studies sampled women who were seeking abortions in health facilities [8, 21, 27], and the remaining 4 studies enrolled women who were attending OB/GYN departments and public health clinics for unstated reasons [7, 20, 23, 29]. Four studies sampled female university students [9, 14, 15, 28]. And one study sampled women through snowballing methodology [19]. In terms of setting, 11 studies were carried out in urban settings [7–9, 12, 14, 18–20, 27, 28, 30], 11 in mixed urban/rural locations [13, 15–17, 21–26, 29], and only 2 studies were based entirely in rural areas [10, 11]. Two of the studies used data from a Demographic and Health Survey (DHS) and a Maternal Health Survey [16, 22] and two were secondary data analyses using the DHS [25, 26].

### Awareness and Knowledge of the legal context of abortion

**Fig 3:**
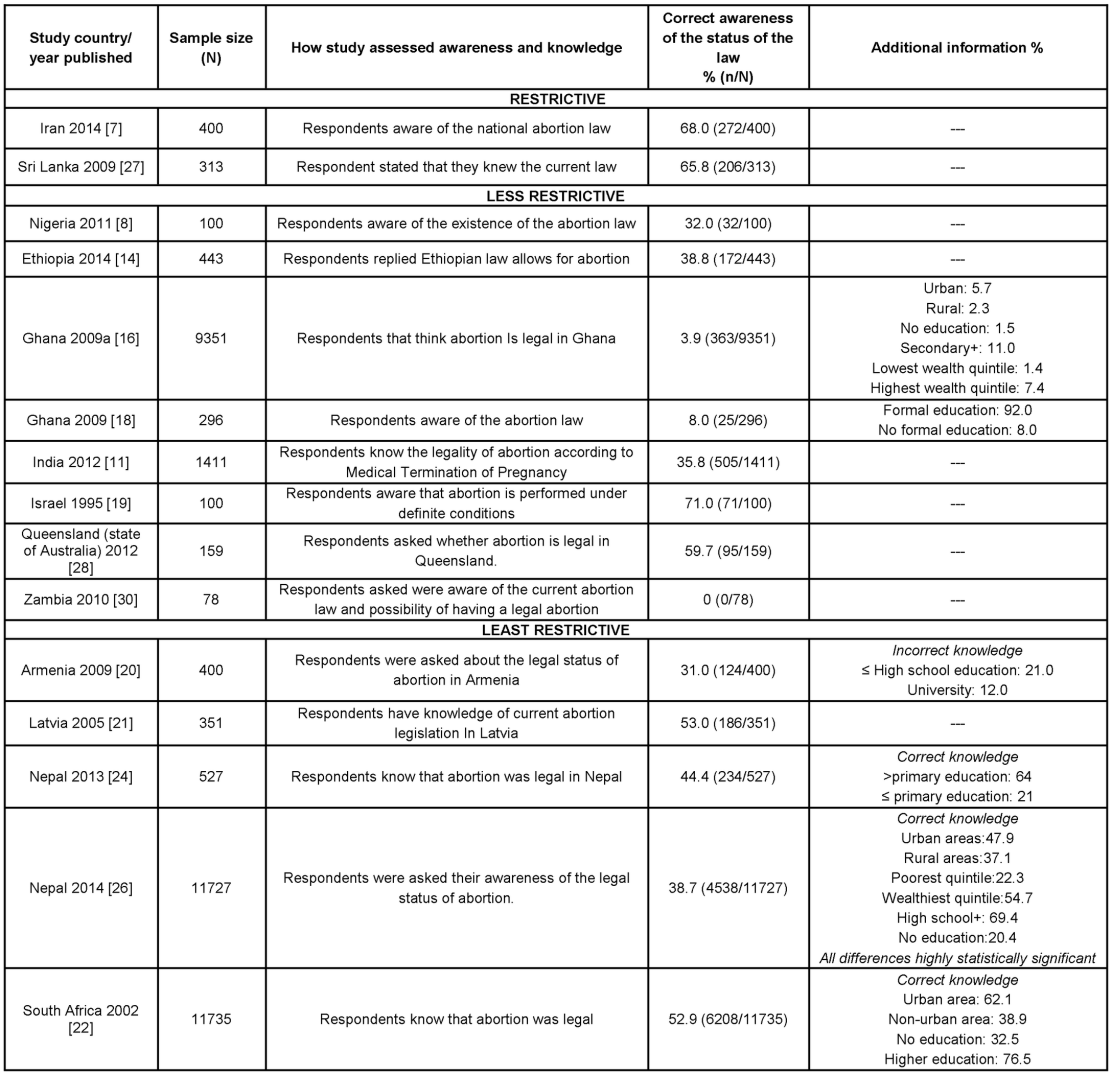
Percentage of participants with correct general awareness and knowledge of abortion law by restrictiveness of legal status (N = 16, 1995–2014).

Women’s correct general awareness and knowledge of the legal status of abortion in their country, as determined by the study researchers, was assessed in 16 studies (Fig 3) [7, 8, 11, 13, 14, 16, 18–22, 24, 26–28, 30]. The proportion of women with correct general awareness and knowledge, ranged between 0% [30] and 71% [19], with general awareness/knowledge being less than 50% in nine of the 16 studies.

Women in the two Ghana studies and the Zambia study had the lowest correct general awareness/knowledge of the legality of abortion in their country [16, 18, 30] ranging from 0% to 8%. In contrast, the study with the highest percentage of correct general awareness/knowledge was from Israel amongst women attending university where 71% of women were aware that abortion is legal when performed under certain conditions [19]. Knowledge was also high at a facility based study in Sri Lanka where of the 313 women attending an abortion clinic, 65.8% stated that they knew the current law [27]. Similarly, 68% of women attending a medical screening clinic in Iran in 2014 had knowledge of the national abortion law [7]. In two other studies that sampled among university students, 38.85% of students in the Ethiopia study correctly responded that the law allowed abortion, and 59% of university students in Queensland also correctly responded that abortion was legal [14, 28].

Armenia and India have had liberal abortion laws for several decades, Armenia since 1955 and India since 1971, however in two studies looking at the women’s general awareness/knowledge of the abortion law show women’s correctly general knowledge was relatively low between 31% and 34% [11, 20].

Data from studies in Armenia, Ghana, India, Nepal, and South Africa indicated that women’s level of general awareness and knowledge varied widely based on their geographical region, wealth and/or education level [13, 16, 20, 22, 24, 26]. Women living in rural regions generally had a lower percentage of correct general awareness/knowledge of their countries’ abortion laws than their urban counterparts, with the greatest differentials seen in South Africa (62.1% in urban vs. 38.9% in rural) [22]. Similar disparities are observed between socioeconomic levels in Nepal study, which reported more than a 30% gap in correct general awareness between the wealthiest and poorest quintiles [26]. Higher levels of education dovetailed greater percentages of correct general awareness and knowledge, though the levels of comparison were highly variable between studies. The starkest differential was seen in the Ghana, where women with some formal education had nearly 85% greater general awareness than women without any formal education (92% vs. 8%) [18] (Fig 3).

#### Awareness and knowledge of changes in national abortion law

**Fig 4:**
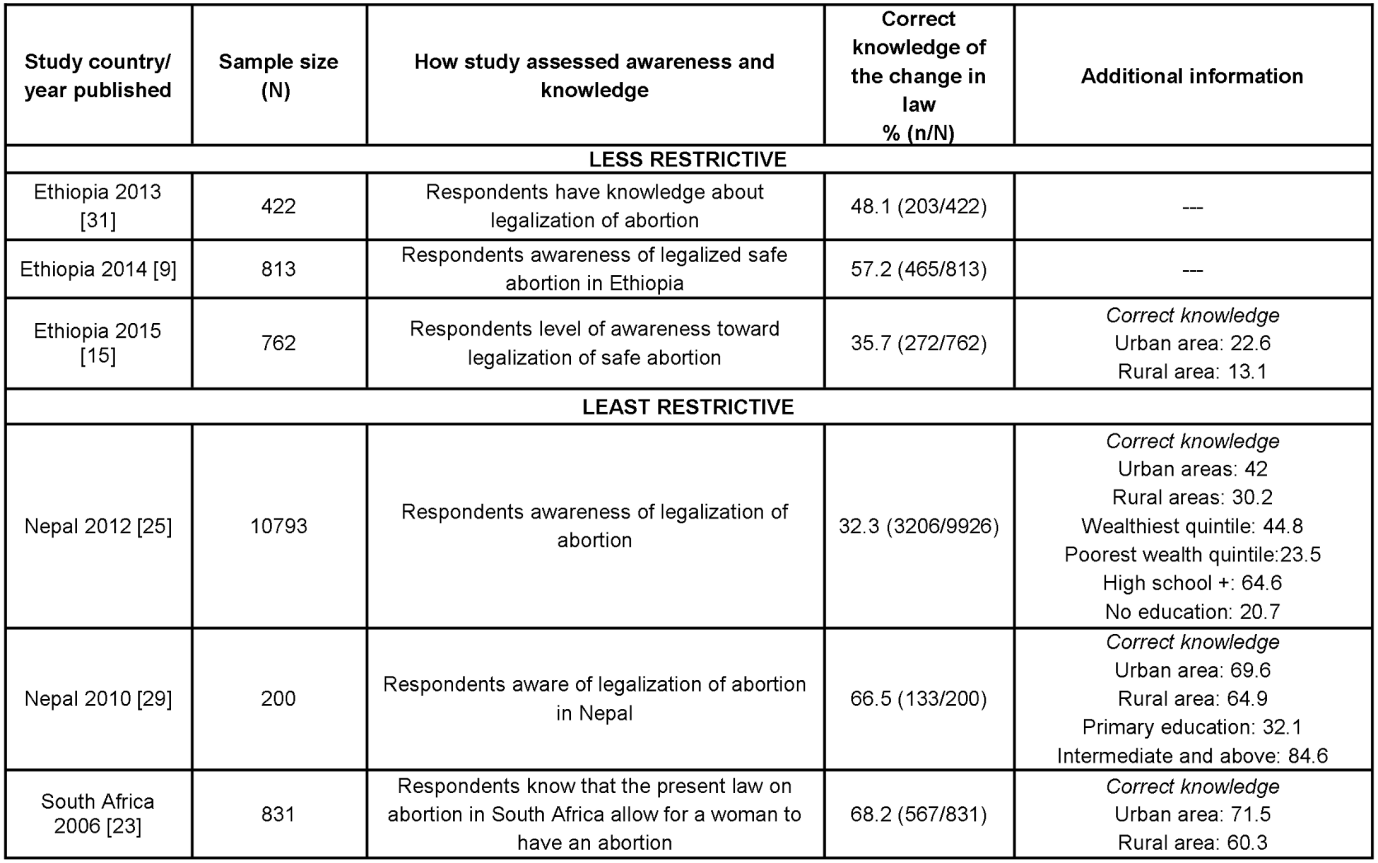
Percentage of participants with correct awareness and knowledge of the legalization/change in abortion law in their country, by restrictiveness of legal status (N = 6, 2006–2015).

Awareness and knowledge of the law in the context of recent liberalisation of the abortion law was reported in six studies. These studies conducted 4 to 8 years after legalization, measured the percentage of women who correctly knew the change in legal status (Fig 4). In South Africa, Nepal, and Ethiopia, where the laws were changed in 1996, 2002, and 2005 respectively, it appears knowledge of the legalization/liberalisation is relatively low, ranging between 32.3% to 68.2% [9, 15, 23, 25, 29, 31]. The proportion of women with correct awareness and knowledge of the law change in Ethiopia reported in three studies conducted 5 to7 years after the legal reform showed no major difference ranging from 35.7–57.2% [9, 15, 31]. Nepal 2012 conducted secondary analyses of DHS data after questions around women’s knowledge of abortion legality were included in the surveys. Similarly, as seen in the previous section, differences in the percentage of correct knowledge existed between socioeconomic strata, once again finding that women living in urban areas and with higher education had higher levels of knowledge than those living in rural areas or with lover levels of education.

### Awareness and knowledge of legal grounds for abortion

**Fig 5:**
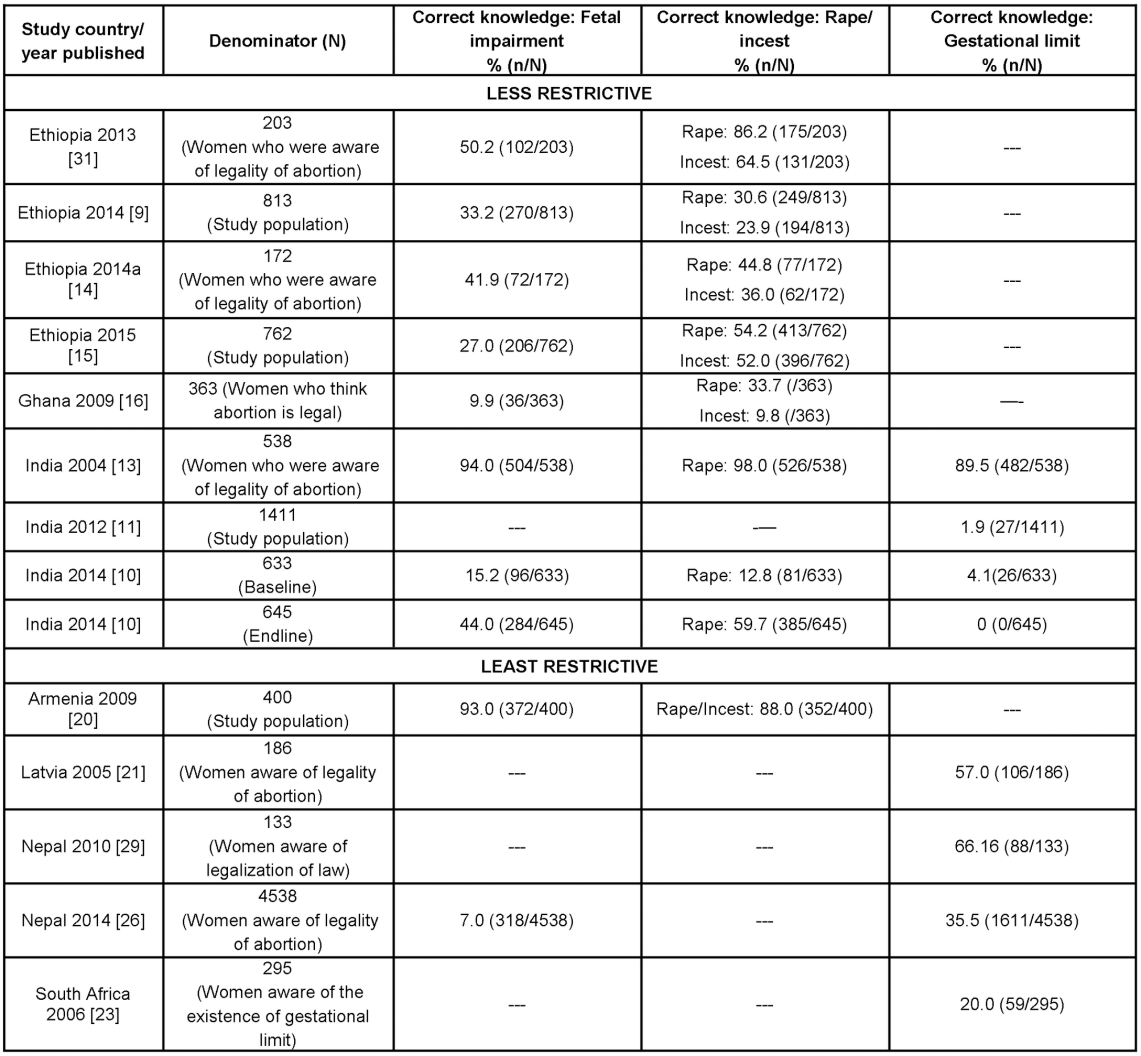
Women's knowledge of legal grounds for abortion and of gestational limits, by restrictiveness of legal status (N = 13, 2004–2015).

Further assessing women’s knowledge of the law by testing whether they were able to identify the specific legal grounds on which abortion was permitted in their respective countries was carried out in thirteen studies from 7 countries [9–11, 13–16, 20, 21, 23, 26, 29, 31]. In this paper, we include information most commonly assessed across studies i.e. awareness of fetal impairment and rape/incest as legal grounds, and knowledge of gestational limits (Fig 5).

According to UNPD, abortion on the basis of rape/incest was permitted in all 4 countries (8 studies) that assessed knowledge of this ground at the time of data collection [6]. Correct knowledge of the rape/incest provision for abortion was generally the highest among the two legal grounds and legal restriction analysed. Correct knowledge of the provision for abortion because of rape ranged from 12.8%–98%, while in the case of incest, knowledge ranged from 9.8%–64.5%. Of the 8 studies that included information on these grounds [9, 10, 13–16, 20, 31], half of the studies reported separate proportions for rape and incest [9, 14, 16, 31]. In Sri Lanka, 17.6% of women incorrectly believed that abortion was allowed when a pregnancy resulted from rape or incest (data not shown) [27].

In the 5 countries (10 studies) that evaluated knowledge on fetal impairment, according to UNPD, abortion on this legal ground was permitted in these countries at the time of the study [6]. For abortion on the grounds of fetal impairment, levels of correct knowledge varied widely from 7% in Nepal study [26] to 93% and 94% in Armenia [20] and in India [13], respectively. In contrast, two studies included in the review reported women’s incorrect knowledge of abortion on the grounds of fetal impairment ranging between 13% and 38% [17, 27].

Women’s correct knowledge of gestational limits ranged from 0% [10] to 89.5% [13]. Both the highest and lowest percentage of knowledge of gestational limits was from studies carried out in India. Of the women in the India 2012 study, 71.1% did not know abortion is legal up to 20 weeks of gestation [11].

Participants overall knowledge of all the grounds that are legal in a country was presented in five included studies (data not shown) [9, 10, 14, 17, 19]. Only 1.9% of women in Ethiopia 2014 study correctly knew all grounds for legal abortion, 4.9% in Ethiopia 2014a study and 29.5% in the Israel 1995 study [9, 14, 19].

A number of studies included in the review included data on women’s incorrect knowledge of the legal regulations in their country besides the ones described above (data not included in figure). Women incorrectly believed that it was illegal for unmarried women to obtain an abortion, ranging from 4.5% in India to 31% in Armenia [11, 13, 20]. In a study from Armenia, a least restrictive setting, 40% of women attending a gynaecological ward erroneously believed that abortion is not allowed on request [20].

## Discussion

This systematic review synthesizes the literature on women’s awareness and knowledge of the abortion law in their own context. Included in this review were 24 studies from 12 different countries.

While study settings and representativeness varied making comparisons difficult, barring a few exceptions, the findings show that correct general awareness and knowledge of the abortion law and legal grounds and restrictions amongst women who participated in these studies was limited, even in countries where the laws were fairly liberal as in India, Zambia or Ghana. Knowledge of liberalisation of laws does not diffuse easily as the two national level studies in Nepal done several years post liberalisation show. In both India and Armenia, knowledge of the legality of abortion was poor even in studies done several decades after liberalisation [10, 11, 13, 17, 20].

In this review, studies that were done in abortion clinics generally reported a higher level of general knowledge than studies done on women presenting to a hospital with a complication from unsafe abortion. Access to and provision of correct information is a key determinant on the pathway to safe abortion. The absence of accurate knowledge and the fear of violating law creates a chilling effect and deter women from seeking health care services [1]. Among women the substantial unmet need for information on the abortion legal context in countries can be the result of the stigmatization of the topic preventing women from seeking information, and/or health care providers personal views on abortion inhibiting an open discussion with women.

The review also highlighted inequities in access to information with knowledge levels being lower among poorer and less educated women in the studies that analysed these factors. Studies included in the review also demonstrated that inequity in wealth, educational level and even difference in geographical location results in the level of women’s correct knowledge to vary amongst these sub-groups of women. Provider’s knowledge of the national abortion legislation and their attitudes towards induced abortions could also be limiting factors in women’s access to information. Women given incorrect information by providers may not realise that it is inaccurate and accept it as fact. Substantial inequality of knowledge existed across sub-groups of women in the studies irrespective of whether they were nationally representative or single facility samples. The inequalities that exist between these population sub-groups within a country, as seen in the included studies, are a well-documented outcome. The recently released *State of Inequality* report highlights and discusses inequalities in knowledge, health access, and health outcomes that exist between these sub-groups within a country and the implication that it has on women’s health [32].

As several studies in the review highlight, interventions to improve the knowledge and understanding of the legal context are feasible. Two studies in the review collected information on where women received information about abortion and legality and outlined four sources: (1) mass media, (2) health personnel, (3) community level groups/activities, and (4) family and friends [11, 29] with media and community sources being the most important. A study in the Jharkhand state of India, demonstrates a successful use of behaviour change communication (BCC) strategies in effectively increasing knowledge and influence positive behavioural change within a study sample [10]. Even a year after the conclusion of the intervention a statistically significant increase in women’s correct knowledge of the abortion law was found and maintained. Two included studies from India indicate that behaviour change communication is an effective implementation method to increase people’s correct knowledge and bring about positive behaviour change [10, 17].

While most of the studies were small and many were facility based and non-representative samples, it was encouraging to see national level data on this included in the Demographic Health Surveys or Maternal Health Surveys in three countries—Nepal in 2006 and 2011, South Africa in 1998 and Ghana in 2009 [22, 25, 26]. There is a greater need for abortion related national level representative data to be collected. Findings from one population group or a single facility study do not allow us to determine whether the findings are representative of what is occurring across the country at a national level.

Women’s own knowledge of the law is only one factor in their being able to access appropriate care. Providers understanding of the laws influences the way they implement these laws in practice. Lack of provisions on how to interpret the abortion law can further reduce a providers understanding of the legal grounds and provisions in which an abortion can be provided. This combined with their hesitance when determining whether a women’s situation meets the legal grounds can result in providers denying a women access to services that she is entitled to [1].

There were no studies from the Latin American region, though language restrictions were not imposed in the search strategy. It is unclear whether there is a lack of studies from this region or that they were missed in this review because we did not systematically search grey literature, unpublished articles and conference reports. Articles from open access publishers that are not indexed in databases searched may also not have been included unless referred to in citations. Demographic and health survey (DHS) databases were not systematically searched, however relevant final reports were included as part of the hand searching of reference lists for the included studies.

## Conclusion

Unsafe abortion remains one of the four main causes of maternal mortality and morbidity. One of the reasons for unsafe abortion is that safe abortion services are frequently unavailable and inaccessible due to a variety of reasons ranging from legal and policy restrictions, lack of accessible and affordable abortion services and lack of knowledge among women regarding the provision of safe abortion. As WHO’s Safe Abortion Guidelines point out the provision of information and knowledge about safe, legal abortion is crucial to protect women’s health and their human rights. UN treaty monitoring bodies require states parties to ensure the provision of information regarding safe abortion services. Women have the right to access full information about the likely benefits and potential adverse effects of proposed procedures and available alternatives.

The findings from this systematic review underscore that across all settings and sub- groups (e.g. legal, urban/rural, least/highly educated, low/high wealth quintiles), women’s understanding of their country’s abortion legal situation appears to be low. As some of the studies show, education and communication interventions can have positive effects on increasing women’s knowledge around the legal context of abortion. Communication methods can be influential on vulnerable population sub-groups in helping increase their awareness and knowledge. Thus, interventions to disseminate accurate information on the legal context are necessary. Knowledge of accessible safe abortion services, providing women with information on the legal context and methods to allow access to such information assist in decreasing the chances that a woman will seek unsafe abortion services and consequently decreasing her likelihood of suffering from abortion related morbidity or mortality.

## Supporting Information

S1 Table Pubmed Search Strategy. (PDF)

S2 Table Prisma 2009 Checklist. (PDF)
