# Supplementary material for: Women’s Awareness and Knowledge of Abortion Laws: A Systematic Review
Source: PLoS One. 2016 Mar 24;11(3):e0152224. doi: 10.1371/journal.pone.0152224 (PMC4807003; doi:10.1371/journal.pone.0152224)
Supplement: S1 Table — (PDF) [file pone.0152224.s001.pdf]

S1 Table– PubMed Search Strategy

#	Searches	Results
1	(((“post-abortion”[Title/Abstract]) OR (“postabortion”[Title/Abstract]) OR (“pregnancy termination”[Title/Abstract) OR (“termination of pregnancy”[Title/Abstract]) OR (“Postconception Fertility Control”[Title/Abstract]) OR (Abortion, legal[MeSH Terms]) OR (“menstrual regulation”[Title/Abstract]) OR (“miscarriage”[Title/Abstract]) or (miscarriage[MeSH Terms]) OR (missed abortion[MeSH Terms]) OR (“abortions”[Title/Abstract]) OR (“abortion”[Title/Abstract]) OR (Abortion, Induced[MeSH Terms])) AND (“1908/01/01”[Date – Publication]: “3000”[Date – Publication]) NOT (“animals”[MeSH Terms] NOT (humans[MESH] AND animals[Mesh])) NOT (“in vitro fertilization”[Title/Abstract]) NOT (“in vitro fertilisation”[Title/Abstract]) NOT (in vitro fertilization[MeSH Terms]) NOT (“threatened abortion”[Title/Abstract])) AND ((Abortion, Legal/Legislation and Jurisprudence[MeSH Terms]) OR (Knowledge[MeSH Terms]) OR (Awareness[MeSH Terms])	700
2	(((“post-abortion”[Title/Abstract]) OR (“postabortion”[Title/Abstract]) OR (“pregnancy termination”[Title/Abstract) OR (“termination of pregnancy”[Title/Abstract]) OR (“Postconception Fertility Control”[Title/Abstract]) OR (Abortion, legal[MeSH Terms]) OR (“menstrual regulation”[Title/Abstract]) OR (“miscarriage”[Title/Abstract]) or (miscarriage[MeSH Terms]) OR (missed abortion[MeSH Terms]) OR (“abortions”[Title/Abstract]) OR (“abortion”[Title/Abstract]) OR (Abortion, Induced[MeSH Terms])) AND (“1908/01/01”[Date – Publication]: “3000”[Date – Publication]) NOT (“animals”[MeSH Terms] NOT (humans[MESH] AND animals[Mesh])) NOT (“in vitro fertilization”[Title/Abstract]) NOT (“in vitro fertilisation”[Title/Abstract]) NOT (in vitro fertilization[MeSH Terms]) NOT (“threatened abortion”[Title/Abstract])) AND ((Law[MeSH Terms]) OR (Knowledge[MeSH Terms]) OR (Awareness[MeSH Terms]))	3545
3	(((“post-abortion”[Title/Abstract]) OR (“postabortion”[Title/Abstract]) OR (“pregnancy termination”[Title/Abstract) OR (“termination of pregnancy”[Title/Abstract]) OR (“Postconception Fertility Control”[Title/Abstract]) OR (Abortion, legal[MeSH Terms]) OR (“menstrual regulation”[Title/Abstract]) OR (“miscarriage”[Title/Abstract]) or (miscarriage[MeSH Terms]) OR (missed abortion[MeSH Terms]) OR (“abortions”[Title/Abstract]) OR (“abortion”[Title/Abstract]) OR (Abortion, Induced[MeSH Terms])) AND (“1908/01/01”[Date – Publication]: “3000”[Date – Publication]) NOT (“animals”[MeSH Terms] NOT (humans[MESH] AND animals[Mesh])) NOT (“in vitro fertilization”[Title/Abstract]) NOT (“in vitro fertilisation”[Title/Abstract]) NOT (in vitro fertilization[MeSH Terms]) NOT (“threatened abortion”[Title/Abstract])) AND ((Law[MeSH Terms]) OR (Legislation[MeSH Terms]) OR (Jurisprudence[MeSH Terms]) OR (Knowledge[MeSH Terms]) OR (Awareness[MeSH Terms]))	5273
4	((((“post-abortion”[Title/Abstract]) OR (“postabortion”[Title/Abstract]) OR (“pregnancy termination”[Title/Abstract) OR (“termination of pregnancy”[Title/Abstract]) OR (“Postconception Fertility Control”[Title/Abstract]) OR (Abortion, legal[MeSH Terms]) OR (“menstrual regulation”[Title/Abstract]) OR (“miscarriage”[Title/Abstract]) or (miscarriage[MeSH Terms]) OR (missed abortion[MeSH Terms]) OR (“abortions”[Title/Abstract]) OR (“abortion”[Title/Abstract]) OR (Abortion, Induced[MeSH Terms])) AND (“1908/01/01”[Date – Publication]: “3000”[Date – Publication]) NOT (“animals”[MeSH Terms] NOT (humans[MESH] AND animals[Mesh])) NOT (“in vitro fertilization”[Title/Abstract]) NOT (“in vitro fertilisation”[Title/Abstract]) NOT (in vitro fertilization[MeSH Terms]) NOT (“threatened abortion”[Title/Abstract])) AND (Knowledge[MeSH Terms])) AND ((Law[MeSH Terms]) OR (Awareness[MeSH Terms]))	7
5	(((“post-abortion”[Title/Abstract]) OR (“postabortion”[Title/Abstract]) OR (“pregnancy termination”[Title/Abstract) OR (“termination of pregnancy”[Title/Abstract]) OR (“Postconception Fertility Control”[Title/Abstract]) OR (Abortion, legal[MeSH Terms]) OR (“menstrual regulation”[Title/Abstract]) OR (“miscarriage”[Title/Abstract]) or (miscarriage[MeSH Terms]) OR (missed abortion[MeSH Terms]) OR (“abortions”[Title/Abstract]) OR (“abortion”[Title/Abstract]) OR (Abortion, Induced[MeSH Terms])) AND (“1908/01/01”[Date – Publication]: “3000”[Date – Publication]) NOT (“animals”[MeSH Terms] NOT (humans[MESH] AND animals[Mesh])) NOT (“in vitro fertilization”[Title/Abstract]) NOT (“in vitro fertilisation”[Title/Abstract]) NOT (in vitro fertilization[MeSH Terms]) NOT (“threatened abortion”[Title/Abstract])) AND (Knowledge[MeSH Terms]) AND ((Law[MeSH Terms]) OR (Legislation[MeSH Terms]) OR (Jurisprudence[MeSH Terms]) OR (Awareness[MeSH Terms]))	32
6	(((“post-abortion”[Title/Abstract]) OR (“postabortion”[Title/Abstract]) OR (“pregnancy termination”[Title/Abstract) OR (“termination of pregnancy”[Title/Abstract]) OR (“Postconception Fertility Control”[Title/Abstract]) OR (Abortion, legal[MeSH Terms]) OR (“menstrual regulation”[Title/Abstract]) OR	0

	(“miscarriage”[Title/Abstract]) or (miscarriage[MeSH Terms]) OR (missed abortion[MeSH Terms]) OR (“abortions”[Title/Abstract]) OR (“abortion”[Title/Abstract]) OR (Abortion, Induced[MeSH Terms])) AND (“1908/01/01”[Date – Publication]: “3000”[Date – Publication]) NOT (“animals”[MeSH Terms] NOT (humans[MESH] AND animals[Mesh])) NOT (“in vitro fertilization”[Title/Abstract]) NOT (“in vitro fertilisation”[Title/Abstract]) NOT (in vitro fertilization[MeSH Terms]) NOT (“threatened abortion”[Title/Abstract])) AND (Knowledge[MeSH Terms]) AND ((Abortion, Legal/Legislation and Jurisprudence[MeSH]) OR (Awareness[MeSH Terms]))	
7	(((“post-abortion”[Title/Abstract]) OR (“postabortion”[Title/Abstract]) OR (“pregnancy termination”[Title/Abstract) OR (“termination of pregnancy”[Title/Abstract]) OR (“Postconception Fertility Control”[Title/Abstract]) OR (Abortion, legal[MeSH Terms]) OR (“menstrual regulation”[Title/Abstract]) OR (“miscarriage”[Title/Abstract]) or (miscarriage[MeSH Terms]) OR (missed abortion[MeSH Terms]) OR (“abortions”[Title/Abstract]) OR (“abortion”[Title/Abstract]) OR (Abortion, Induced[MeSH Terms])) AND (“1908/01/01”[Date – Publication]: “3000”[Date – Publication]) NOT (“animals”[MeSH Terms] NOT (humans[MESH] AND animals[Mesh])) NOT (“in vitro fertilization”[Title/Abstract]) NOT (“in vitro fertilisation”[Title/Abstract]) NOT (in vitro fertilization[MeSH Terms]) NOT (“threatened abortion”[Title/Abstract])) AND (Knowledge[MeSH Terms]) OR ((Abortion, Legal/Legislation and Jurisprudence[MeSH]) OR (Awareness[MeSH Terms]))	14038
8	(((“post-abortion”[Title/Abstract]) OR (“postabortion”[Title/Abstract]) OR (“pregnancy termination”[Title/Abstract) OR (“termination of pregnancy”[Title/Abstract]) OR (“Postconception Fertility Control”[Title/Abstract]) OR (Abortion, legal[MeSH Terms]) OR (“menstrual regulation”[Title/Abstract]) OR (“miscarriage”[Title/Abstract]) or (miscarriage[MeSH Terms]) OR (missed abortion[MeSH Terms]) OR (“abortions”[Title/Abstract]) OR (“abortion”[Title/Abstract]) OR (Abortion, Induced[MeSH Terms])) AND (“1908/01/01”[Date – Publication]: “3000”[Date – Publication]) NOT (“animals”[MeSH Terms] NOT (humans[MESH] AND animals[Mesh])) NOT (“in vitro fertilization”[Title/Abstract]) NOT (“in vitro fertilisation”[Title/Abstract]) NOT (in vitro fertilization[MeSH Terms]) NOT (“threatened abortion”[Title/Abstract])) AND ((Abortion, Legal/Legislation and Jurisprudence[MeSH Terms]) OR (Knowledge[MeSH Terms]) OR (Knowledge[Title/Abstract]) OR (Awareness[MeSH Terms]))	2565 *Chosen search strategy*
